# Treatment of chronic hepatitis C in Croatian war veterans: experiences from Croatian reference center for viral hepatitis

**DOI:** 10.3325/cmj.2011.52.35

**Published:** 2011-02

**Authors:** Neven Papić, Snježana Židovec Lepej, Ivan Kurelac, Vjeran Čajić, Jelena Budimir, Davorka Dušek, Adriana Vince

**Affiliations:** 1 Department of Viral Hepatitis, University Hospital for Infectious Diseases, Zagreb, Croatia; 2 Department of Molecular Diagnostics and Cellular Immunity, University Hospital for Infectious Diseases, Zagreb, Croatia

## Abstract

**Aim:**

To examine the risk factors, comorbidity, severity of liver disease, treatment course, and outcome in Croatian war veterans with chronic hepatitis C, especially those suffering from posttraumatic stress disorder (PTSD).

**Methods:**

We collected medical records of 170 adult men diagnosed with chronic hepatitis C who started treatment with a combination of pegylated interferon-alpha and ribavirin between January 2003 and June 2009 at the Croatian Reference Centre for Viral Hepatitis.

**Results:**

Participants’ mean age was 43 ± 9 years. Among 170 participants, there were 37 war veterans (22%). The main risk factor in veteran patients were operative procedures with transfusions (46% vs 5% in non-veterans; *P* < 0.001) and in non-veteran patients intravenous drug use (42.1% vs 13%; *P* < 0.001). The average duration of infection was longer in war veterans (14.5 ± 3.4 vs 12.2 ± 7.2 years; *P* = 0.020). The percentage of PTSD comorbidity in the whole group was 11% (18/170) and in the war veterans group 49% (18/37). The prevalence of sustained virological response in patients with PTSD was 50% and in patients without PTSD 56%. Treatment reduction in patients with PTSD (33%) was higher than in patients without PTSD (12%;*P* = 0.030).

**Conclusion:**

Croatian war veterans are a group with high risk of chronic hepatitis C infection because many of them were wounded during the Croatian War 1991-1995. Considerations about PTSD as a contraindication for interferon treatment are unjustified. If treated, patients with PTSD have an equal chance of achieving sustained virological response as patients without PTSD.

Infectious diseases have historically been responsible for the majority of war-related deaths; however, medical and military advances have reversed this trend and today military health care is confronted with specific medical issues related to war veterans’ social, psychological, and epidemiological characteristics. Chronic hepatitis C (CHC) has in the recent years been identified as the single most important emerging pathogen in the war veterans’ health care system.

Military veterans are at risk of acquiring viral hepatitis through training and combat (field bleeding, surgery, and transfusions). Several studies conducted in the USA have shown a higher prevalence of hepatitis C virus (HCV) infection in veterans than in the general population (1-4). The US Department of Veteran Affairs reported a 3-fold prevalence of HCV seropositivity in war veterans (6.6%) than in the general population (2%) (1,3).

Effective treatment of CHC with pegylated interferon-alpha (PEG-IFN-α) and ribavirin, as measured by sustained virological response (SVR), eradicates the virus in 40%-50% of patients infected with HCV genotype 1 and approximately 80% of patients with genotypes 2 or 3 (5,6). Even with major advances in the treatment of CHC, only a minority of HCV-infected people (up to 30%) are prescribed treatment in the real-life setting. Butt et al (2) reported a treatment prescription rate of 11.8% in 113 927 US veterans with CHC. The major barriers to the initiation of antiviral therapy were high rates of psychiatric comorbidities, substance use, and standard well-established predictors of poor virological response (male sex, alcohol abuse, overweight, and older age).

Significant proportion of veterans experiences moderate to severe depressive symptoms. El-Serag et al (7) showed that 33.5% of 33 824 US veterans with CHC were diagnosed with posttraumatic stress disorder (PTSD). Although recent studies suggested that psychiatric disorders should not be considered as an absolute contraindication for interferon-based therapy, these patients are less likely to initiate and successfully complete the treatment (8-10). CHC treatment can be compromised by a variety of neuropsychiatric symptoms such as fatigue, irritability, depression, anxiety, and psychosis, which either limit the administered doses of drugs or lead to a discontinuation of treatment, reducing the probability of achieving SVR (5,6,11).

During the Croatian War 1991-1995, there were approximately 12 000 killed and 35 000 wounded. Out of 30 520 wounded (7163 civilians and 23 351 soldiers), 84.5% were surgically treated, mostly before 1993, ie, before the routine introduction of blood donor anti-HCV screening in Croatia (12). The reported prevalence of PTSD in Croatian war veterans ranges between 16% and 34% (13,14), but no systematic studies on HCV seroprevalence in Croatian war veterans have been performed so far. Consequently, HCV infection and CHC in this neglected population remain an important public health issue.

The primary aim of this study was to examine the risk factors, comorbidity, and severity of liver disease, treatment course, and SVR in Croatian war veterans compared with the general male population. As PTSD is recognized as the most important comorbidity among war veterans, the second aim was to compare the course of PEG-IFN-α and ribavirin treatment in patients with PTSD and patients without PTSD.

## Participants and methods

### Participants

This retrospective study included 170 adult men diagnosed with CHC who started treatment with a combination of PEG-IFN-α and ribavirin between January 2003 and June 2009 at the Croatian Reference Centre for Viral Hepatitis, Ministry of Health and Social Welfare of the Republic of Croatia and Department of Viral Hepatitis, University Hospital for Infectious Diseases (UHID), Zagreb, Croatia. The mean age of the patients was 43 ± 9 years and weight was 82 ± 9 kg. The group comprised 37 war veterans (21.7%). The prevalence of PTSD comorbidity was 10.6% in the whole group (18/170) and 48.6% (18/37) among war veterans. All patients with PTSD were receiving psychiatric care at baseline (before the initiation of antiviral therapy) and throughout the study period. They were diagnosed with combat-related PTSD by their current psychiatric clinician (according to the 10th revision of the International Classification of Diseases). A total of 125 patients were treated with Peg-IFN-α2a 180 μg/week + ribavirin and 45 were treated with Peg-IFN-α2b 1.5 μg/week + ribavirin. Ribavirin was administered in the recommended doses, according to weight. The duration of therapy in HCV genotypes 1 and 4 was 48 weeks, and in genotypes 2 and 3 was 24 weeks. The therapy was discontinued in patients with genotype 1 and genotype 4 if viral load decreased by less than 2 log HCV RNA copies/mL at week 12 compared with baseline values and if HCV RNA was still detectable at week 24.

### Method and definitions

Croatian war veterans and general male population were compared according to demographic data, risk factors, genotypes, duration of infection, and type of pegylated interferon. The patients were classified into three groups: war veterans, war veterans with PTSD, and male population without PTSD. Patients who had SVR and who discontinued treatment or required a reduction of therapeutic doses with and without PTSD were also compared.

SVR was defined as undetectable HCV RNA at 24 weeks after the end of antiviral therapy. HCV RNA quantification was performed by COBAS Ampliprep/COBAS TaqMan HCV test (Roche Diagnostics, Diagnostic Systems, Pleasanton, CA, USA). HCV genotyping was performed by VERSANT HCV Genotyping assay (LIPA, Bayer Diagnostics, Puteaux, Cedex, France) at the Department of Molecular Diagnostics and Cellular Immunity, UHID. Liver biopsy was performed at the Department of Viral Hepatitis, UHID and the Ishak scoring system (15) was used as an indicator of histological activity (stages of fibrosis from 0-6, where 0 represents no fibrosis and 6 represents established cirrhosis).

### Data collection

The study was approved by the Ethics Committee of the UHID. Records of all men treated at the Department of Viral Hepatitis, UHID during the study period were extracted and used for collection of clinical and laboratory data. Virological results obtained from patient records were double-checked at the database of the Department of Molecular Diagnostics and Cellular Immunity, UHID. Liver biopsy was performed at the UHID, and histological analysis was performed by a skilled pathologist at the Zagreb University Hospital. Liver biopsy data were double-checked at the database of the Department for Viral Hepatitis, UHID. The patients underwent psychiatric evaluation in order to assess their eligibility for CHC treatment, as required by the Croatian Institute for Health Insurance. Data on PTSD comorbidity was collected from psychiatry medical records. Disease duration was calculated from the date of blood transfusion before 1993, the date of severe operation, or the time of the drug injection that was the onset of infection.

### Statistical analysis

The baseline demographic, clinical, and laboratory data were evaluated and presented descriptively. Fisher exact test and Wilcoxon rank sum test were used to compare the two groups as appropriate. Reduction rate, cessation of therapy, and SVR were compared using χ^2^ and Fisher exact test as appropriate. Statistical analysis was performed using Prism statistical software, version 5.0, (GraphPad Software, San Diego, CA, USA). All comparisons were two tailed, and *P* < 0.05 was considered significant.

## Results

### Patient characteristics

A total of 170 patients were included in the study. War veterans and participants from the general male population showed similar demographic, clinical, and virological characteristics (Table 1).

**Table 1:** Baseline characteristics of 170 patients with chronic hepatitis C (37 veteran and 133 non-veteran)

	No. (%) of war veterans	No. (%) of non-veterans	*P*
Age, years	45 ± 8	42 ± 11	0.041*
Weight, kg	80 ± 16	82 ± 11	0.699*
Risk factor:			
unknown	5 (13.5)	35 (26.3)	0.127**^†^**
operation	7 (8.1)	5 (3.7)	0.005^†^
transfusion	1 (2.7)	25 (18.7)	0.018^†^
operation and transfusion	17 (45.9)	7 (5.3)	<0.001^†^
intravenous drug use	5 (13.5)	56 (42.1)	<0.001^†^
wounding	2 (5.4)	3 (2.2)	0.298^†^
sex		2 (1.5)	
Genotype:			
1	25 (67.6)	92 (69.2)	0.843^†^
2	1 (2.7)	1 (0.7)	0.389^†^
3	10 (27.0)	38 (28.6)	>0.950^†^
4	1 (2.7)	2 (1.5)	0.524^†^
Liver biopsy (Ishak score, mean ± standard deviation):^‡^			
fibrosis	3.44 ± 1.05	3.45 ± 1.06	0.884*
histology activity score	8.81 ± 3.37	8.28 ± 2.90	0.586*
Duration of infection, years:	14.5 ± 3.38	12.2 ± 7.17	0.020*
Treatment:			
pegylated interferonα2a	30 (81.1)	95 (71.4)	0.295^†^
pegylated interferonα2b	7 (18.9)	38 (28.5)	0.295^†^

*Wilcoxon rank sum test.

**†**Fisher exact test.

‡Ishak et al (15).

There were significant differences between the two groups in transmission routes. The main risk factor among war veterans were operative procedures with transfusions (45.9% vs 5.3%; *P* < 0.001, Fisher exact test) and among non-veterans intravenous drug use (42.1% vs 13.5%; *P* < 0.001). Duration of infection was longer among war veterans (14.5 ± 3.3 vs 12.2 ± 7.1 years; *P* = 0.020, Wilcoxon rank sum test). Both groups had similar genotype, liver biopsy result, age, weight, and type of pegylated interferon (Table 1).

### Reduction and discontinuation of therapy

The therapy was reduced or discontinued in 155 of the 170 patients. The remaining patients had unavailable or incomplete medical records.

Dose reduction was more frequent in patients without PTSD than in those with PTSD (Table 2). There were no significant differences in discontinuation of therapy, although patients with PTSD had somewhat more frequent discontinuations.

**Table 2:** Treatment modification and sustained virological response (SVR) in participants with posttraumatic stress disorder (PTSD) (N = 18) and without it (N = 137 for treatment modification, 152 for SVR)

	No. (%) of patients		
	with PTSD	without PTSD	P*
Treatment modification	6 (33.3)^†^	17 (12.4)^‡^	0.030
Pegylated interferon-α dose reduction	2 (11.1)	8 (5.8)	0.327
Ribavirin dose reduction	3 (16.7)	5 (3.6)	0.051
Pegylated interferon-α and ribavirin dose reduction	1 (5.5)	4 (2.9)	0.465
Treatment discontinuation	3 (16.6)	10 (7.3)	0.178
Sustained virological response (intention-to-treat analysis)	9 (50.0)	87 (57.2)	0.619
Lost to follow-up	1 (5.5)	9 (5.9)	>0.950

*Fisher exact test.

^†^Reasons for treatment modification: anemia or/and neutropenia, n = 3; depression, n = 2; psychosis, n = 1.

^‡^Reasons for treatment modification: anemia, neutropenia or/and thrombocytopenia, n = 7; depression, n = 4; prolonged rash and itch, n = 2; hypothyroidism, n = 1; non-safety reasons, n = 3.

The main reasons for discontinuation were depression and severe psychiatric disorders that required hospitalization. The main reasons for dose reduction were severe anemia, leukopenia, thrombocytopenia, anxiety, and depression symptoms that were not satisfactorily resolved with antidepressants.

### Virological response in patients with and without PTSD

Analyses included data from all patients who received at least one dose of medication (intention to treat analysis). In the whole group of CHC patients, SVR was achieved in 96 out of 170 patients (56.4%). SVR rate for genotype 1 and 4 was 47% and for genotype 2 and 3 was 74%. In the subgroup of war veterans, 21 of 37 patients (56.7%) achieved SVR.

In order to determine the influence of PTSD comorbidity on virological response, several clinical parameters were compared between patients with PTSD and without PTSD and no significant differences were found (Table 2). However, 4 patients with PTSD (22.2%) did not finish the protocol, 3 for safety reasons due to serious side effects and 1 due to non-safety reasons (loss to follow-up). In the group of patients without PTSD, 22 (14.5%) did not finish the protocol; 10 due to adverse effects, 3 due to poor virological response, and 9 due to loss to follow-up.

## Discussion

To our knowledge, this is the first study describing the outcome of CHC treatment in war veterans in Croatia. War veterans represented 21% of the male population receiving therapy from January 2003 to July 2009 at the UHID, accounting for a significant part of HCV-infected population. The main risk factors were major operation with transfusions and intravenous drug use, which is the major transmission route among the general male population.

Croatian War 1991-1995 brought considerable military casualties (16). The majority of approximately 23 000 wounded soldiers were operated on several times with transfusion, exposing them to substantial CHC risk (12,17). According to the World Heath Organization, the estimated prevalence of HCV infection in Croatia is 1.4%, however it is higher among war veterans and other war casualties (18,19).

High risk of CHC was first recognized in Vietnam War veterans from the USA. However, they differed considerably from the participants of this study – were older and had more psychiatric, alcohol, and drug abuse disorders, and lower virological responses (1,3,4,20). Other studies on USA veterans have also reported different results than our study. Butt et al (21) reported the completion rate of 22.5% among 16 043 veterans-in-care, compared with 83% among patients with PTSD in our study. Nguyen et al (20) found intravenous drug use to be the most common risk factor in the USA war veterans, 60% of whom had psychiatric diagnoses. The incidence of PTSD in the USA HCV-infected war veterans was 19%-34% (7,20), which is similar tour study (48.6%). This is also similar to earlier Croatian studies that have reported up to 34% prevalence of PTSD or PTSD with a comorbid psychiatric diagnosis among Croatian war veterans (13,14).

War veterans with psychiatric disorder have been denied treatment for HCV infection, mainly because interferon treatment may worsen the underlying psychiatric illness, resulting in premature discontinuation of antiviral treatment and non-compliance (8,9), especially since PTSD is a disorder linked with substantial distress and dysfunction. CHC has been found to provoke exacerbation of PTSD and, together with interferon, cause hyperarousal and re-experiencing of symptoms, thus complicating the course of treatment (8,11).

Our study revealed a higher rate of treatment modification and discontinuation in patients with PTSD than in patients without PTSD, mainly due to psychiatric disorders, which finally resulted in a slightly lower SVR in patients with PTSD than in patients without PTSD. However, according to previously published evidence (8-10) and this study, the majority of patients with PTSD can be treated safely and effectively, and have an equal chance to achieve SVR as patients without PTSD. Furthermore, our war veterans mainly stopped treatment for safety reasons, exhibited better compliance than general male population, and only a minority missed the follow-up 24 weeks after the end of treatment.

The main limitation of this study is a retrospective nature of the collected data and a limited number of patients. However, this was the maximum number of patients that we could have collected since the study was performed in the largest treatment center for HCV-positive patients in Croatia. Most of the data from medical charts were double-checked in available databases, which reduced potential biases caused by lacking or incomplete documentation. Furthermore, all patients met the eligibility criteria for treatment according to the Croatian Institute for Health Insurance, resulting in more detailed medical charts than usual.

This is the first report on HCV infection among Croatian war veterans, the true prevalence of which in Croatia is still unknown, and further screening and public health programs are necessary to elucidate the true scope of this problem. The high rate of PTSD comorbidity requires the development of multidisciplinary treatment models that would provide coordinated mental health and medical care for patients with CHC. Finally, a successful antiviral treatment is needed to decrease the transmission of HCV in the general population.

In summary, Croatian war veterans have a high risk of CHC, especially those with PTSD comorbidity. Our data showed that these patients had an equal chance of achieving SVR, and therefore PTSD comorbidity should not be considered an exclusion criterion for interferon treatment.

## References

[1] Briggs ME, Baker C, Hall R, Gaziano JM, Gagnon D, Bzowej N (2001). Prevalence and risk factors for hepatitis C virus infection at an urban Veterans Administration medical center.. Hepatology.

[2] Butt AA, Justice AC, Skanderson M, Rigsby MO, Good CB, Kwoh CK (2007). Rate and predictors of treatment prescription for hepatitis C.. Gut.

[3] Dominitz JA, Boyko EJ, Koepsell TD, Heagerty PJ, Maynard C, Sporleder JL (2005). Elevated prevalence of hepatitis C infection in users of United States veterans medical centers.. Hepatology.

[4] Hyams KC, Riddle J, Rubertone M, Trump D, Alter MJ, Cruess DF (2001). Prevalence and incidence of hepatitis C virus infection in the US military: a seroepidemiologic survey of 21,000 troops.. Am J Epidemiol.

[5] McHutchison JG, Lawitz EJ, Shiffman ML, Muir AJ, Galler GW, McCone J (2009). Peginterferon alfa-2b or alfa-2a with ribavirin for treatment of hepatitis C infection.. N Engl J Med.

[6] Rumi MG, Aghemo A, Prati GM, D'Ambrosio R, Donato MF, Soffredini R (2010). Randomized study of peginterferon-alpha2a plus ribavirin vs peginterferon-alpha2b plus ribavirin in chronic hepatitis C.. Gastroenterology.

[7] el-Serag HB, Kunik M, Richardson P, Rabeneck L (2002). Psychiatric disorders among veterans with hepatitis C infection.. Gastroenterology.

[8] Cawthorne CH, Rudat KR, Burton MS, Brown KE, Luxon BA, Janney CG (2002). Limited success of HCV antiviral therapy in United States veterans.. Am J Gastroenterol.

[9] Rifai MA, Moles JK, Short DD (2006). Hepatitis C treatment eligibility and outcomes among patients with psychiatric illness.. Psychiatr Serv.

[10] Tavakoli-Tabasi S, Rowan P, Abdul-Latif M, Kunik ME, El-Serag HB (2005). Utility of a depression score to predict candidacy for hepatitis C virus therapy in veterans: a prospective longitudinal study.. Aliment Pharmacol Ther.

[11] Maunder RG, Hunter JJ, Feinman SV (1998). Interferon treatment of hepatitis C associated with symptoms of PTSD.. Psychosomatics.

[12] Hebrang A, Henigsberg N, Golem AZ, Vidjak V, Brnic Z, Hrabac P (2006). Care of military and civilian casualties during the war in Croatia. Acta Med Croatica.

[13] Gregurek R, Pavic L, Vuger-Kovacic H, Potrebica S, Bitar Z, Kovacic D (2001). Increase of frequency of post-traumatic stress disorder in disabled war veterans during prolonged stay in a rehabilitation hospital.. Croat Med J.

[14] Kozaric-Kovacic D, Bajs M, Vidosic S, Matic A, Alegic Karin A, Peraica T (2004). Change of diagnosis of post-traumatic stress disorder related to compensation-seeking.. Croat Med J.

[15] Ishak K, Baptista A, Bianchi L, Callea F, De Groote J, Gudat F (1995). Histological grading and staging of chronic hepatitis.. J Hepatol.

[16] Kuzman M, Tomic B, Stevanovic R, Ljubicic M, Katalinic D, Rodin U (1993). Fatalities in the war in Croatia, 1991 and 1992. Underlying and external causes of death.. JAMA.

[17] Horzic M, Bunoza D, Maric K, Poje A (1996). A prospective study of the application of transfusions in a field hospital during the war in Croatia.. Mil Med.

[18] Hepatitis C. – global prevalence (update). In: Weekly Epidemiological Report. 74 ed. Geneva: World Health Organization; 1999.

[19] Cavlek TV, Margan IG, Lepej SZ, Kolaric B, Vince A (2009). Seroprevalence, risk factors, and hepatitis C virus genotypes in groups with high-risk sexual behavior in Croatia.. J Med Virol.

[20] Nguyen HA, Miller AI, Dieperink E, Willenbring ML, Tetrick LL, Durfee JM (2002). Spectrum of disease in U.S. veteran patients with hepatitis C.. Am J Gastroenterol.

[21] Butt AA, McGinnis KA, Skanderson M, Justice AC (2010). Hepatitis C treatment completion rates in routine clinical care.. Liver Int.

